# Prize Essays

**Published:** 1872-12-01

**Authors:** 


### PRIZE ESSAYS.

The Committee of the American Medical Editors' Association, appointed to consider and make known a proper subject for prize essays, have recommended the following for the prize to be awarded in May, 1873 : "*The Pathology and Treatment of Diseases of the Ovaries.*" And for the prize to be awarded in May, 1874, the following : "*At what stages of Pulmonary Tuberculosis is a Change of Climate Desirable ; what are the Principles which should govern us in choosing the kind of change to be made ; and the best localities in North America to send Patients of this class.*" The prize offered is $100, and competition is open to all medical *editors*. It is not open to all physicians.—*Nashville Journal of Medicine and Surgery.*

We answer the closing question of the above paragraph, by saying that the action of the "Association of American Medical Editors" restricted competition for the prizes offered, to medical editors alone. The object evidently was, to encourage both investigation and writing among those connected with our medical periodicals. And we hope the time will not pass without developing a lively competition.

PHILADELPHIA MEDICAL TIMES.—This valuable periodical, published by J. B. Lippincott & Co., and hitherto issued semi-monthly, is now published *weekly*.

STRYCHNIA IN TYPHOID FEVER.—A correspondent in Wisconsin, writes to the *Medical and Surgical Reporter*, of Philadelphia, thus: 'I am not aware that, strychnia, or nux vomica is used in typhoid fever; but having witnessed its curative action in dysentery, I was induced to give it a trial, and am satisfied that it is one of the best remedies we possess for supporting the patient and restraining the excessive action of the bowels." It is quite possible that the *Reporter's* correspondent may not be aware of the fact, and yet it is true, that strychnia has been used and its value well known in the treatment of certain stages of typhoid fever for a quarter of a century at least. It was used and strongly recommended by Dr. Thomas Spencer, of the Geneva Medical College, more than twenty-five years since. And it has been used both in hospital and private practice at least twenty years in this city, and repeated references made to it in our medical journals.

